# Genetic aetiology of self-harm ideation and behaviour

**DOI:** 10.1038/s41598-020-66737-9

**Published:** 2020-06-16

**Authors:** Adrian I. Campos, Karin J. H. Verweij, Dixie J. Statham, Pamela A. F. Madden, Dominique F. Maciejewski, Katrina A. S. Davis, Ann John, Matthew Hotopf, Andrew C. Heath, Nicholas G. Martin, Miguel E. Rentería

**Affiliations:** 1 Department of Genetics & Computational Biology, QIMR Berghofer Medical Research Institute, Brisbane, QLD 4006 Australia; 2 Faculty of Medicine, The University of Queensland, Herston, QLD Australia; 3 Department of Psychiatry, Amsterdam UMC, University of Amsterdam, Meibergdreef 9, 1105 AZ Amsterdam, the Netherlands; 4 Discipline of Psychology, School of Health and Life Sciences, Federation University, Ballarat, VIC 3550 Australia; 5 Department of Psychiatry, Washington University School of Medicine, St Louis, MO 63110 USA; 6 Department of Developmental Psychopathology, Behavioural Science Institute, Nijmegen, the Netherlands; 7 KCL Institute of Psychiatry, Psychology and Neuroscience, London, UK; 8 South London and Maudsley NHS Foundation Trust, London, UK; 9 HDRUK, Swansea University Medical School, Swansea, UK

**Keywords:** Behavioural genetics, Emotion

## Abstract

Family studies have identified a heritable component to self-harm that is partially independent from comorbid psychiatric disorders. However, the genetic aetiology of *broad sense* (non-suicidal and suicidal) self-harm has not been characterised on the molecular level. In addition, controversy exists about the degree to which suicidal and non-suicidal self-harm share a common genetic aetiology. In the present study, we conduct genome-wide association studies (GWAS) on lifetime *self-harm ideation* and *self-harm behaviour* (i.e. any lifetime self-harm act regardless of suicidal intent) using data from the UK Biobank (n > 156,000). We also perform genome wide gene-based tests and characterize the SNP heritability and genetic correlations between these traits. Finally, we test whether polygenic risk scores (PRS) for *self-harm ideation* and *self-harm behaviour* predict *suicide attempt*, *suicide thoughts* and *non-suicidal self-harm* (NSSH) in an independent target sample of 8,703 Australian adults. Our GWAS results identified one genome-wide significant locus associated with each of the two phenotypes. SNP heritability (*h*_*snp*_^2^) estimates were ~10%, and both traits were highly genetically correlated (*LDSC* r_g_ > 0.8). Gene-based tests identified seven genes associated with *self-harm ideation* and four with *self-harm behaviour*. Furthermore, in the target sample, PRS for *self-harm ideation* were significantly associated with *suicide thoughts* and *NSSH*, and PRS for *self-harm behaviour* predicted *suicide thoughts* and *suicide attempt*. Follow up regressions identified a shared genetic aetiology between NSSH and suicide thoughts, and between suicide thoughts and suicide attempt. Evidence for shared genetic aetiology between NSSH and suicide attempt was not statistically significant.

## Introduction

Every year nearly one million people take their own lives^1^, making suicide a pressing issue of considerable social and economic burden. Moreover, self-harm behaviours are now recognized by the American Psychiatric Association as independent conditions for further study. Namely, *non-suicidal self-injury* and *suicidal behaviour disorder* were recently introduced in the section 3 of the Diagnostic and Statistical Manual of Mental Disorders (DSM-V)^2^. The lifetime prevalence estimate for suicide thoughts is ~10%, while suicide attempt and non-suicidal self-harm (NSSH) affect ~2.5 and ~5% of the population, respectively^3–5^. Higher rates have been reported amongst children and adolescents^6^. The key difference between suicidal and non-suicidal self-harm is that the former implies an intent to die as a consequence of the act. Non-suicidal self-harm acts include equally dangerous behaviours such as cutting, burning or poisoning, but are underlined by a different motivation such as seeking attention or the desire to feel pain.

Twin and family studies indicate that NSSH, suicide thoughts and suicide attempt are moderately heritable^7,8^. Multiple studies have documented that the presence of a psychiatric disorder considerably increases the risk for both suicidal and non-suicidal self-harm^9–11^ but a sizeable genetic component (33-51% of variance) of suicide risk is not explained by underlying psychiatric conditions^12–16^. Importantly, controversy exists about whether NSSH and suicide attempt are part of the same liability spectrum^7,16–20^. Notably, twin studies have identified a genetic correlation between suicide thoughts and NSSH^7^, but the extent to which shared genetic factors underlie NSSH, suicide thoughts and suicide attempt (i.e. a *self-harm liability continuum hypothesis*) still remains elusive. Investigating *broad sense* self-harm could improve our understanding of the aetiology and underpinnings of the liability to both suicidal and non-suicidal self-harm.

Although several genome-wide association studies (GWAS) on suicidality have been published to date^21–31^, robustly associated genetic variants are still elusive^32^. A number of studies assessing the genetic predictability of suicidality have suggested a shared aetiology between depression and suicidality, and suggestive evidence for suicidality-related associations^32–35^. Investigating the genetic architecture of self-harm regardless of suicidal intent, could be valuable to gain biological insights into the relationship between self-harm and suicide. In the present study, we explore the genetic aetiology of *lifetime self-harm ideation* and *lifetime self-harm behaviour* (regardless of suicidal intent) using a GWAS and PRS approach. An overview of the phenotypes and terminology used throughout this manuscript is available in Table 1.

**Table 1:** Overview of the phenotypes and terminology in this study.

Behaviours (lifetime)	Suicidal ideation	NSSH ideation	NSSH	Suicide attempt
PRS categories	Self-harm ideation		Self-harm (behaviour)	
UKB	Undifferentiated n = 23192 (14.7%). No lifetime suicidal ideation available in UKB		Undifferentiated n = 6872 (4.4%)	
			N for NSSH only = 3089 (NSSH > 2.0%)	N = 3563 (2.3%)
Outcome categories	Thoughts of suicide	NA	NSSH	Suicide attempt
Queensland Twin Registry	27.10%	NA	3.20%	4.00%

## Methods

### Discovery sample

The discovery sample consisted of ~156,700 participants from the UK Biobank (as of February 2019) with self-harm data (lifetime history of *self-harm ideation* and *self-harm behaviour*)^36^. Self-harm was assessed as described previously^37^. DNA extraction and genotyping are described in ref. ^38^. Notably, genotyping was performed using two highly related arrays: the UK BiLEVE Axiom array and the UK Biobank Axion Array.

Briefly, the self-harm behaviour item was “Have you deliberately harmed yourself, whether or not you meant to end your life?” (No = 150,008, yes = 6,872). The self-harm ideation item was: “Have you contemplated harming yourself (for example by cutting, biting, hitting yourself or taking an overdose)?” (No = 133,524, yes = 23,192). Notably, both these phenotypes make no distinction between suicidal and non-suicidal self-harm. As such, they include both suicidal and non-suicidal self-harm. A liability threshold model (tetrachoric correlation; psych package in R) estimated the traits to be highly correlated ρ = 0.89 (95% c.i. 0.88–0.89; N reporting both ideation and behaviour = 6446).

### GWAS

We conducted two GWAS, on lifetime *self-harm ideation* and lifetime *self-harm behaviour*. Association analyses were performed using BOLT_LMM^39^, based on a linear mixed model and allele dosages (of the effect allele based on imputed data) accounting for the first 20 genetic ancestry principal components, standard covariates (age, age^2^, sex, sex*age as fixed effect predictors), and correcting for cryptic relatedness and population stratification using a genetic relatedness matrix as the random effects variance covariance structure. Approximately 48,000 individuals of non-European ancestry were excluded from the analyses. A stringent, but standard quality-control (QC) protocol^40^ was applied: variants with low minor allele count (MAC < 25), low quality imputation (INFO < 0.8) or with a deviation from the Hardy-Weinberg equilibrium (HWEp < 1e-10) were excluded from further analyses.

### Gene-based test analyses

Gene-based association analysis was conducted for *self-harm ideation* or *self-harm behaviour* using MAGMA^41^ as implemented on the FUMA web platform^42^. Briefly, SNPs were mapped to ~20,000 protein coding genes based on their genomic location. Then, the independent SNP association statistics were combined to yield gene-based mean χ2 statistics. Genome-wide significance level was defined as 2.652e-6 (Bonferroni corrected alpha <0.05).

### PRS target sample

The target sample consisted of individuals from two cohorts of the Queensland Twin Registry. Individuals were recruited and participated in structured telephone or paper interviews assessing psychiatric disorders, substance abuse and living conditions. Detailed information on the cohorts has been published previously^43,44^. Items from the Semi-Structured Assessment for the Genetics of Alcoholism (SSAGA) assessing self-harm behaviours were included in both cohorts, a detailed description of the items and their application is available in^34^. Briefly, participants were first asked whether they experienced any suicidal thoughts, then suicide attempt, and finally acts of self-harm not related to suicide attempts. The items used to determine lifetime prevalence of self-harm behaviours were: *“Have you ever thought about taking your own life?”; “Have you ever tried to take your own life?”* and *“(Other than when you tried to take your own life) Did you ever hurt yourself on purpose, for example, by cutting or burning yourself?”*.

The genotyping and quality control (QC) protocol for the target sample have been described previously^34,45^. Briefly, standard protocols for DNA collection and extraction were used. Genotyping was carried out using commercial Illumina SNP arrays. Platform specific QC was performed including: Hardy Weinberg equilibrium deviation, individual SNP call rates, minor allele frequency threshold, and removal of population outliers (i.e. non-European ancestry as determined by principal component analysis). Genotype data were imputed using the haplotype reference consortium (HRC) reference panel.

### SNP heritability and genetic correlation

The amount of variance in risk explained by SNP effect sizes (i.e. the SNP heritability or h_snp_^2^) was calculated using LD-score regression as previously described^46^. The software *ldsc* v 1.0.0 was used to calculate both the h_snp_^2^ and the genetic correlation between both GWAS summary statistics. This method relies on the relationship between the non-centrality parameter (NCP or χ^2^) of GWAS results and LD scores (the sum of LD-r^2^ of a SNP against all other SNPs on the same population) expected under a polygenic assumption (the bigger the LD score, the more likely to tag a causal variant)^47^. The fact that the expected value of the NCP for a given SNP is a function of the genetic covariance (h_g_^2^) of the trait and the LDscore of that SNP, allow us to estimate the SNP based heritability^46^ (h_snp_^2^) and co-heritability (r_g_)^48^ of a set of traits given their summary statistics and known LD patterns. As the population prevalence of *broad sense* self-harm behaviours on the UK has not been reported, we assumed the population prevalence to be equal to the discovery sample prevalence when transforming to the liability scale using *ldsc* and thus estimates of h_snp_^2^ should be referred to with caution. Genetic correlations between the traits under study and other traits and diseases (~760 traits) were explored using the LD-Hub web platform^46,49^. We used a stringent definition of a significant genetic correlation (FDR < 0.01 using a Benjamini-Hochberg multiple testing correction, for a list of traits see Supplementary Data 1).

### Polygenic risk scores and prediction analysis

To calculate the genetic predisposition (risk) of our target sample to the traits of interest, each variant’s effect size was obtained from the GWAS summary statistics. Our PRS estimation pipeline excluded *indels*, strand ambiguous- and low (R^2^ < 0.6) imputation quality-variants. The most significant independent SNPs were selected using a conservative clumping procedure (PLINK1.9; p1 = 1, p^2^ = 1, r^2^ = 0.1, kb=10000)^50^ to correct for inflation arising from linkage disequilibrium (LD). Eight different PRS were calculated for each individual using different p-value thresholds (p < 5 × 10^−8^, p < 1 × 10^−5^, p < 0.001, p < 0.01, p < 0.05, p < 0.1, p < 0.5, p < 1) as criteria for SNP inclusion on the PRS calculation. PRS were calculated using a dosage assumption, therefore multiplying the effect size of a given SNP by the imputed number of copies (using dosage probabilities) of the effect allele present in an individual. Finally, the SNP dosage effects were summed across all loci per individual.

To assess the association between the genetic liability to *self-harm behaviour* and *self-harm ideation* (i.e. the PRS) with actual self-harm phenotypes in the target sample, we employed a linear mixed model regression framework. Briefly, the PRS was added to the model as a predictor variable while accounting for sex, age, age^2^, sex*age, the first five genetic principal components and imputation run, an *in-house* set of variables that capture array and cohort differences^34,51^, as fixed effects. Correcting for relatedness is crucial when examining family cohorts. Given varying degrees of relatedness in our sample, we employed a linear mixed model using genetic restricted maximal likelihood (GREML) with a random effects variance covariance structure defined by the sample’s genetic relatedness matrix obtained from GCTA 1.91.7^52,53^. This method has been previously used to deal with related individuals in related target samples of PRS studies^51,54^. A partial R^2^ was used to estimate the variance explained by the PRS using the formula:

1 $${R}^{2}={\left(\frac{\beta }{{\sigma }_{pheno}}{\sigma }_{PRS}\right)}^{2}$$

where *β* represents the PRS fixed effect estimate, ${\sigma }_{pheno}$ the standard deviation of the phenotype and ${\sigma }_{PRS}$ the standard deviation of the PRS respectively. Statistical significance threshold was defined accounting for multiple testing using a matrix spectral decomposition approach^55,56^ to estimate the number of effective variables being tested. The final significance threshold was defined at α < =0.0064

As sensitivity analysis, we tested whether the liability to major depressive disorder was driving polygenic prediction. To this end, MDD-PRS were calculated using SBayesR^57^ based on available summary statistics leaving out the Australian sample^58^. The *self-harm behaviour* (and *self-harm ideation*) phenotype associations were reproduced with MDD-PRS as a covariate to identify the variance explained by *self-harm behaviour* (or *self-harm ideation*) over and above the effect of MDD liability.

Further, we were interested on whether the prediction of our PRS on the traits (Suicide attempt, suicide thoughts and NSSH) was due to shared or independent genetic factors. To this end, the significant associations between the PRS for *self-harm ideation* and suicide thoughts were reproduced including NSSH as a covariate. Likewise, the association between PRS for *self-harm ideation* and NSSH was reproduced with suicide thoughts as a covariate. The same approach was used for the *self-harm behaviour* PRS but using either suicide thoughts or suicide attempt (the phenotypes with some evidence of association) as a covariate. If the significant PRS-phenotype associations were driven through the same genetic components, adding one of the phenotypes as a covariate should implicitly capture the shared genetic predisposition, thus removing the observed association. Any residual prediction would imply that independent genetic factors, captured by our GWAS, are underlying each phenotype.

## Results

### Sample demographics and self-harm behaviours prevalence

The demographic composition for both the discovery and target samples are given in Tables 2 and 3 respectively. In the discovery sample, where genetic correlates of *broad sense self-harm ideation* and *self-harm behaviour* were assessed, males and females presented a similar age range, but females showed a higher prevalence of both *self-harm ideation* and *self-harm behaviour*. Prevalence of NSSH, suicide thoughts and suicide attempt on the target sample were 3.4, 26.4 and 3.8% respectively. Prevalence of suicide thoughts was slightly lower in the female subgroup while suicide attempt was higher (Table 3).

**Table 2:** Discovery sample (UK-Biobank) demographics and prevalence of self-harm behaviours.

	Age (SD)	Self-harm ideation (%*)	Self-harm behaviour (%*)
Total sample	55.9 (7.7)	23,192 (14.8)	6872 (4.4)
Males	56.6 (7.8)	7,951 (11.7)	2102 (3.1)
Females	55.5 (7.7)	15,241 (17.2)	4770 (5.4)

*Percentage estimated based only on the amount of non-missing subjects for each phenotype.

**Table 3:** Target sample (Queensland Twin Registry) demographics and prevalence of self-harm behaviours (genotyped individuals only).

	N	Age (SD)^t^	SA (%)	ST (%)	NSSH (%)
Total	8703	42.8 (12.3)	335 (3.8)	2296 (26.4)	181 (3.4*)
M	3407	43.0 (12.0)	100 (2.9)	951 (27.9)	76 (3.3*)
F	5296	42.7 (12.4)	235 (4.4)	1345 (25.4)	105 (3.5*)

SA - suicide attempt, ST - suicide thoughts, NSSH - non-suicidal self-harm. *Percentage estimated based only on non-missing values due to a cohort missing the NSSH item. ^t^Ages at the time of survey.

### GWAS of broad sense self-harm behaviours

**Figure 1:**
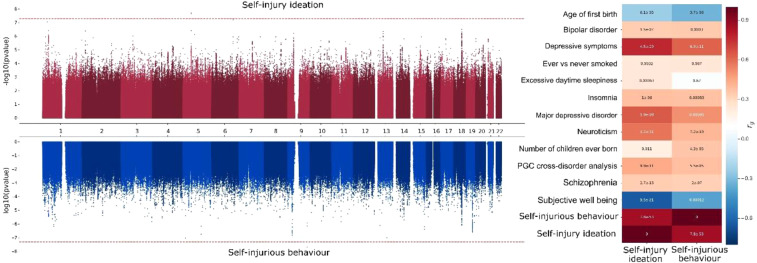
GWAS results and genetic correlations of broad sense self-harm thoughts and behaviours. Miami plot (left panel) depicts the genome-wide association results for the phenotypes studied. The x-axis represents the genomic position, while the y axis represents the significance of the association between each SNP and the phenotype; the top panel represents significance as –log10 (pvalue), while the bottom panel uses log10 (pvalue), in both cases, the farther from the x axis (middle) line, the more significant the association between the phenotype and the variant. On the right side, a heat map depicts the genetic correlations (rg) between published trait GWAS and our GWAS for self-harm ideation or self-harm behaviour. Only traits with a Benjamini-Hochberg fdr <0.01 for at least one phenotype and generated using studies independent from the UK-Biobank are depicted here (All the results, including UK-B traits, are available in Supplementary Data 1).

**Figure 2:**
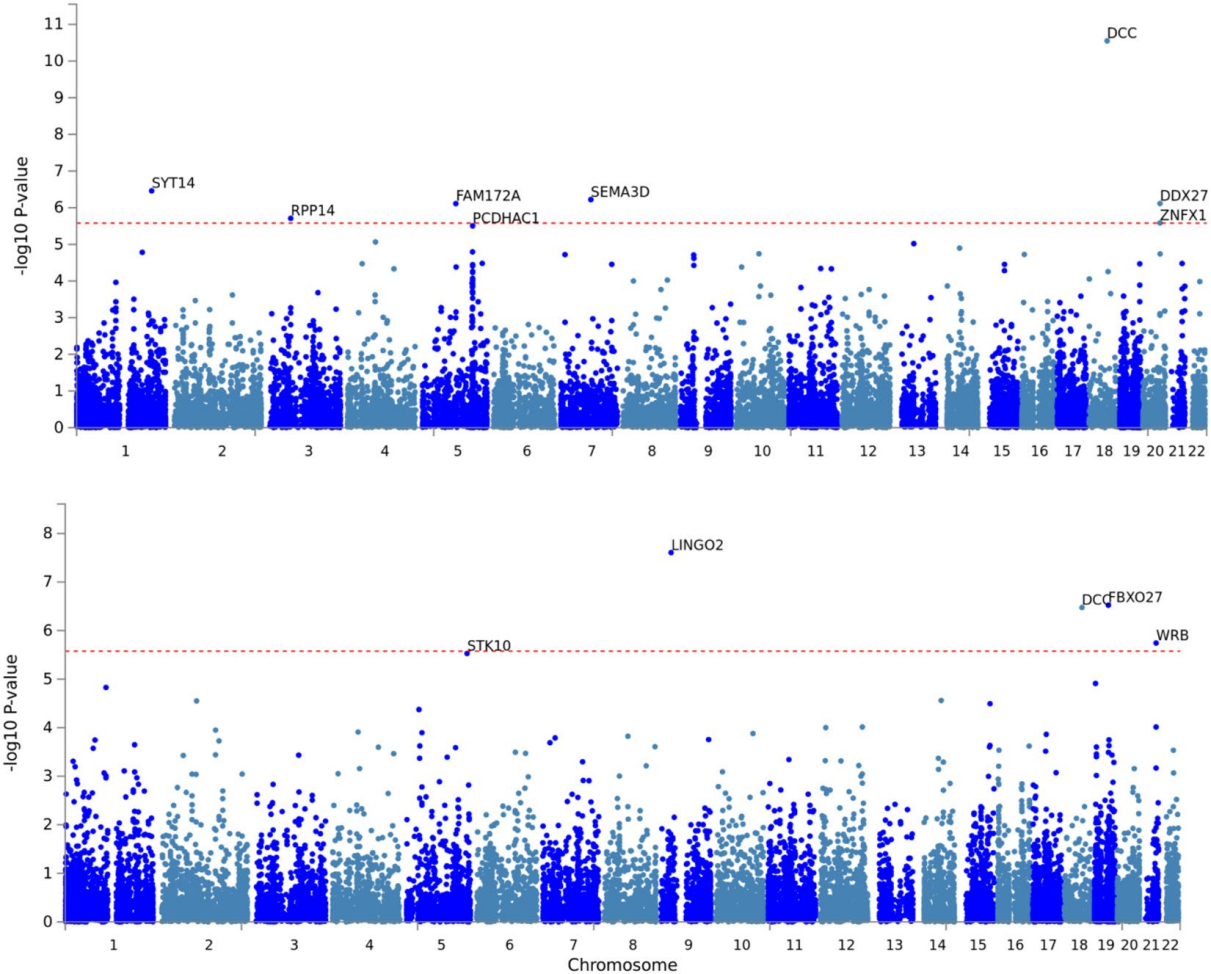
Gene based association. Manhattan plots depicting gene-based test results of the GWAS. The x-axis represents the genes genomic position, and the y axis the significance (−log_10_(p-value)) of the association between the genes and the studied phenotype. The phenotypes for the top and bottom panels are self-harm ideation and self-harm behaviour respectively.

Two GWAS assessing *self-harm ideation* and *self-harm behaviour* were performed. After QC, the GWAS for *self-harm ideation* identified one genome-wide significant locus on chromosome five. The GWAS for *self-harm behaviour* presented one genome-wide significant hit on chromosome nine (Table 4 and Fig. 1). A gene-based association test identified seven significantly associated genes with *self-harm ideation*: *SYT14*, *RPP14*, *FAM17*2*A*, *SEMA3D*, *DCC*, *DDX27* and *ZNFX1*. For the GWAS on *self-harm behaviour*, four genes *LINGO2*, *DCC*, *FBXO27* and *WRB* showed an association surpassing genome-wide significance (Fig. 2).

**Table 4:** Variants associated with either self-harm ideation or self-harm behavior.

SNP	CHR	BP	Effect allele	Other allele	Self-Harm ideation beta (p-value)	Self-Harm behaviour beta (p-value)
rs4865733	5	51819679	T	C	−0.008 (1.90E-08)	−0.003 (7.50E-05)
rs7721698	5	51821771	C	T	−0.008 (2.20E-08)	−0.003 (7.40E-05)
rs567805973	9	122489021	C	T	−0.035 (0.014)	−0.046 (2.10E-08)

SNP – Single nucleotide polymorphism; CHR- chromosome; BP- Base position.

### Heritability and genetic correlation of broad sense self-harm behaviours and thoughts

The SNP heritability (*h*_*snp*_^*2*^) on the liability scale for both traits was estimated to be 11.1% (SE = 1.7%) for *self-harm behaviour* and 10.1% (SE = 1.0%) for *self-harm ideation*. Further, the traits were highly genetically correlated with each other (*r*_g_ = 0.85, p = 7.8e^−53^). High genetic correlations with psychiatric disorders such as anxiety, depression and schizophrenia, symptoms such as insomnia, and personality traits such as irritability, miserableness, mood swings, and risk-taking, among others, were identified for both traits (Fig. 1). Furthermore, a negative correlation with subjective well-being and age at first birth (i.e. the age in which a person has their first child) was observed (Fig. 1 and Supplementary Fig. 1). As expected from their high genetic correlation, the genetic correlations of *self-harm ideation* and *self-harm behaviour* across a range of available traits were highly similar (R > 0.8, Supplementary Fig. 2).

### Polygenic risk score prediction

**Figure 3:**
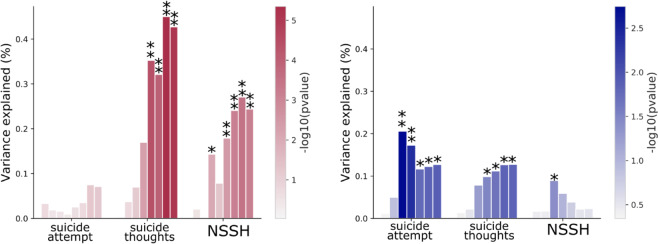
Polygenic prediction of self-harm behaviours. Bar plots represent the amount of variance explained by the polygenic risk scores on the self-harm phenotypes. The red colour (left side) shows the associations of the PRS for self-harm ideation whereas the blue colour (right side) depicts the associations of the PRS for self-harm behaviour. For each phenotype studied, the amount of variance explained by a PRS including variants with an increasingly liberal p-value threshold (from left to right) is shown. The bars are ordered based on the p-value cut-off used to construct the PRS (increasingly liberal p-values). The height of each bar represents the amount of variance explained. The p-value for the association between the PRS and the phenotype is shown with a colour scale. *Represents p < 0.05; **represents significant after multiple testing correction).

We calculated PRS on an independent sample of ~8,700 Australian adults. A summary of the PRS variables and results is available on Supplementary Tables S1 and S2. The PRS for *self-harm ideation* significantly predicted suicide thoughts (maximum variance explained 0.45%, p = 4.5e^−6^) and NSSH (maximum variance explained 0.27%, p = 3.6e^−4^) (Fig. 3). Notably, the associations were significant for PRS including variants with p value cut-offs <0.001 (or less stringent cut-offs) for suicide thoughts, and p < 0.01 (or less stringent cut-offs) for NSSH. The PRS for *self-harm behaviour* predicted suicide attempt (maximum variance explained = 0.20%) and suicide thoughts (maximum variance explained = 0.13%) (Fig. 3). Although the PRS for *self-harm behaviour* did not predict NSSH in our sample, the PRS for *self-harm behaviour* was predictive of broad sense self-harm (regardless of suicidal intent; maximum variance explained >0.30% p < 0.001). This association was also diminished when correcting for NSSH and further diminished when correcting for suicide attempt (Supplementary Fig. 3).

### Sensitivity analyses

Given the high genetic correlation identified with depression, we performed a sensitivity analysis to assess whether depression specific genetic factors were driving the polygenic prediction. To this end, PRS for MDD were calculated (see methods), and the associations described above were reproduced but including MDD-PRS as a covariate. The overall variance explained was reduced. Nonetheless, there was still evidence for an association between *self-harm behaviour* PRS and suicide attempt or suicide thoughts; and between *self-harm ideation* PRS and suicidal thoughts and non-suicidal self-harm (Supplementary Fig. 4).

**Figure 4:**
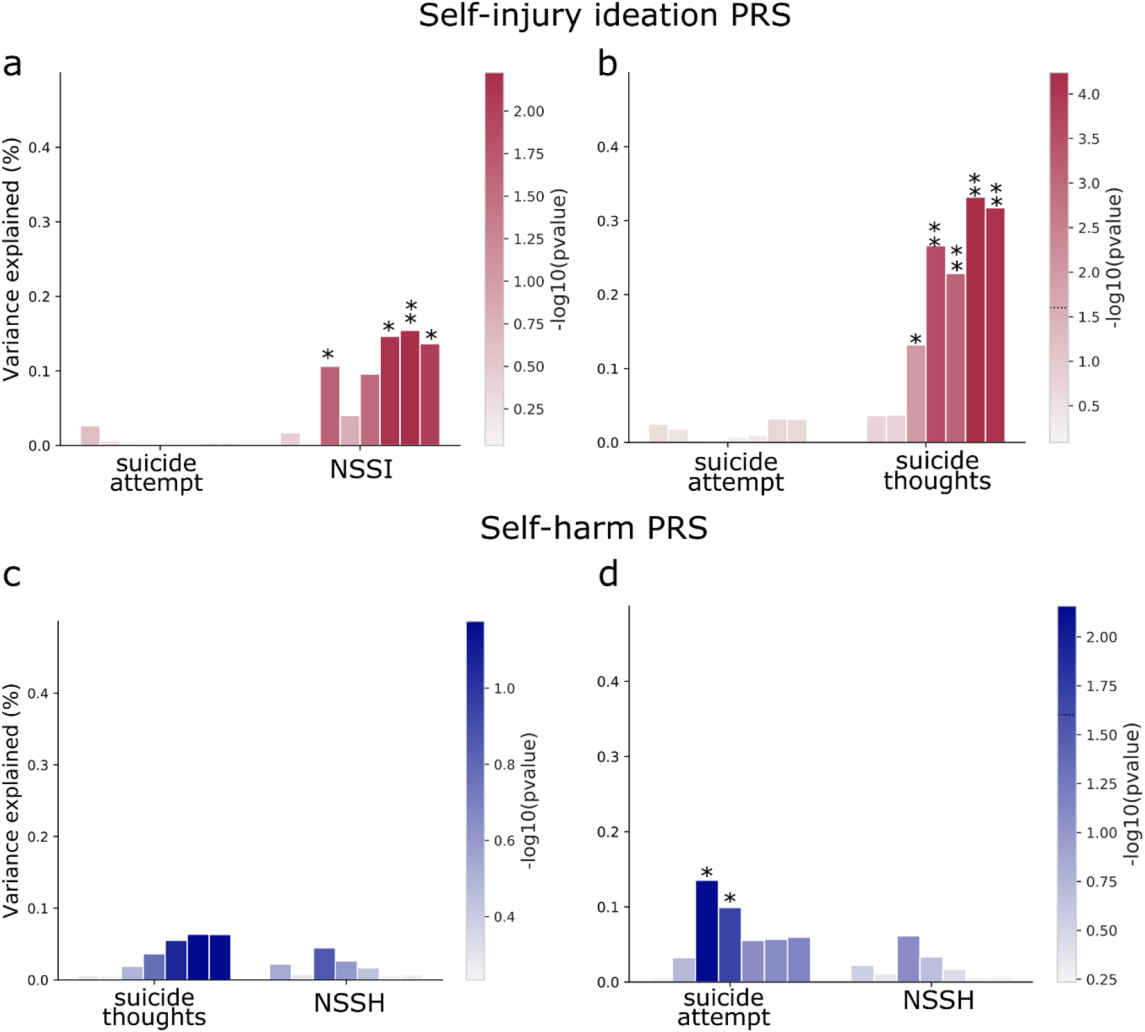
Assessing the shared genetic aetiology of self-harm behaviours. The PRS-phenotype associations were repeated but accounting for the other significantly associated phenotype as a covariate (see methods). Panel (a) shows the result of PRS for self-harm ideation while accounting for suicide thoughts, (**b**) PRS for self-harm ideation while accounting for NSSH, (**c**) PRS for self-harm behaviour while accounting for suicide attempt and (**d**) PRS for self-harm behaviour while accounting for suicide thoughts. *p < 0.05, **significant after multiple testing correction.

We also performed secondary analyses to test whether the associations between PRS and phenotypes were driven by shared genetic factors (see methods). The PRS for *self-harm ideation* significantly predicted suicide thoughts after including NSSH as a covariate; it also predicted NSSH after including suicide thoughts as a covariate, albeit with a smaller proportion of variance explained (~0.33% and ~0.15 respectively; Fig. 4). The PRS for *self-harm behaviour* continued to significantly predict suicidal attempts after including suicide thoughts as a covariate, but the association with suicide thoughts disappeared when suicidal attempt was included as a covariate in the model (Fig. 4).

## Discussion

We explored the genetic architecture of *broad sense self-harm* by performing GWAS of *self-harm ideation* and *self-harm behaviour* in a population-based sample. The identification of only one genome-wide significant locus for each phenotype suggests that better powered genetic studies of self-harm are needed, and indicate that these traits are highly polygenic. Given the self-reported nature of the phenotypes, it is possible that recall bias and differences in subjective understanding of the mental health items could increase the noise to signal ratio in the discovery GWAS. Self-harm is a complex behaviour that encompasses subtypes with varying severities and recurrence rates^59^, which are not captured by the single item used to ascertain self-harm on the UKB.

We also identified eleven genome wide significant genes using gene-based association tests. Seven were associated with *self-harm ideation* and four with *self-harm behaviour*. The gene with the strongest association with *self-harm ideation*, and the only gene associated with both phenotypes, was *DCC*. *DCC* is a gene involved in prefrontal cortex innervation and development. This observation could be consistent with reports of structural abnormalities on the brains of suicidal subjects^60,61^. Consistent with our results, DCC has been independently linked to suicidal severity on the UK-B^62^ and there is evidence of elevated *DCC* expression in the prefrontal cortex of *post-mortem* brains of subjects that died by suicide^63^.

Regarding genes associated with *self-harm ideation*, *FAM172A* has been previously associated with differential methylation linked to childhood stress in girls^64^, and is located in a locus recently associated with insomnia^65^, a phenotype known to be associated with self-harm and suicidality^66,67^. Notably, previous studies suggest that the gene *SEMA3D*, known to be associated with schizophrenia^68,69^, could also be associated with suicidality^24^. A link between *SYT14* and bipolar disorder has been reported^70^. *DDX27* has been associated with intelligence^71,72^, and a study reporting a relationship between lower IQ and suicide attempt has been published^73^. Variants near *DDX27* and *ZNFX1* have been nominally linked to proneness to anger^74^. No obvious relationship between *RPP14* and any psychiatric or behavioural phenotype has been reported in GWAS databases or the literature. Notably, we identified suggestive associations of a cluster of protocadherin genes (*PCDH*) on chromosome 5. *PCDHAC1* is enriched in serotonergic cells in mice^75^ and *PCDH*-family differential methylation has been recently associated with early-onset major depression^76^ and previously associated with schizophrenia, bipolar disorder^77^ and autism^78^.

Three genes: *LINGO2, FBXO27* and *WRB* were associated with *self-harm behaviour*. *LINGO2* and its paralog *LINGO1* have been linked to neurodegenerative and psychiatric disorders^79,80^. Notably, previous results suggest that differential methylation on the promoter of *FBXO27* might be linked with childhood physical aggression^81^, which is known to be highly associated with suicidal behaviours^82^. While *WRB* has been linked to cognitive impairment, it is unclear how it relates to *self-harm behaviour*. Finally, we found evidence for a suggestive association between *STK10* and *self-harm behaviour*. *STK10* has been linked with childhood cognitive ability^83^ and observed to be hyper-hydroxy-methylated upon acute stress^84^. Altogether these results provide promising candidate genes associated with *self-harm ideation* and behaviour. Future analyses should focus on replicating these findings and assessing their underlying mechanistic and possible translational roles on self-harm.

While the SNP-heritability of *self-harm ideation* and behaviour was significant—and some proportion of variance on the studied phenotypes was explained by PRS—, the percentage of variance explained was still far from the heritability estimates for NSSH (h^2^~37–59%), suicidal ideation (h^2^~47–66%) and suicide attempt (h^2^~55%) reported in twin and family studies^7,15,85^. Although the UKB recruitment process does not represent a random sample of the UK population^86^—and there is evidence of genetic factors associated with completion of the mental health section^87^— our PRS results provide evidence that the genetic associations discovered have some predictive power (albeit still a small one) over self-harm related phenotypes on an independent population. The above observations call for novel, well powered genetic studies of self-harm which will be required in order to obtain accurate SNP effect sizes^88^. A recent study suggest that even after well powered GWA studies have been conducted, most of the missing heritability for a phenotype is tagged by variants with a low MAF that cannot be easily imputed^89^. Therefore, whole genome sequencing studies of self-harm and suicidality could be paramount to achieve a complete understanding of the genetic architecture underlying self-harm.

The fact that a PRS for self-harm thoughts was able to explain up to 0.27% of the variance of NSSH is consistent with a previous twin study reporting a significant co-heritability between suicide ideation and NSSH^7^. Further, the follow up regressions correcting for suicide thoughts when predicting NSSH, and for NSSH when predicting suicide thoughts showed a reduction of the amount of variance explained. This observation is consistent with a partial shared genetic aetiology between them. To the best of our knowledge, this is the first study to date to report on a positive genetic prediction of NSSH. Notably, a previous PRS study assessing depression and self-harm in our sample^34^ identified an association between the genetic predisposition for depression and suicidal ideation, but no robust association with suicide attempt or non-suicidal self-harm. Thus, two possible explanations exist: (i) the previously stated genetic^7^ and phenotypic^90^ correlations between suicide thoughts and NSSH should be explained by depression-independent genetic factors, or (ii) the study by Maciejewski *et al*. (2017) was underpowered, possibly due to the low accuracy of the available summary statistics at that time and the number of cases in the target population, which limited its ability to detect an association between NSSH and the MDD-PRS.

Our results support the existence of a genetic overlap between NSSH and suicide thoughts. We also detected genetic overlap between suicide thoughts and suicide attempt, which was evidenced by the association between the PRS for *self-harm behaviour* and suicide thoughts disappearing after correcting for suicide attempt. Finally, evidence of a shared genetic aetiology between suicide attempt and NSSH did not reach statistical significance, as no PRS simultaneously (robustly) predicted both of them. A possible explanation is that genetic predisposition to NSSH is not associated with suicide attempt predisposition, in spite of the known overlap between NSSH and suicidality^11,91^. Another plausible explanation is a lack of power either on the discovery (due to either number of cases or a high noise to signal ratio) or target samples to detect an overlap between NSSH and suicide attempt due to a low prevalence of both behaviours.

Self-harm behaviours are likely to share genetic aetiology with several other traits and disorders. We have detected some of these candidate traits, such as neuroticism, nervousness and a risk-taking personality by assessing their genetic correlation with self-harm thoughts and behaviours. Interestingly, a negative genetic correlation of both self-harm phenotypes with *age at first birth* was detected, and this was also observed for the most recent GWAS on depression^58^. Moreover, another study identified a negative correlation between maternal age at child birth and suicidality. Although the design of this study corrected for genetic confounding^92^, the association would support the hypothesis that genetic factors predisposing to changes in maternal age could impact on depression and therefore on suicidality and self-harm. The usage of PRS for independent traits to predict self-harm results in a tool of great power to test for pleiotropy and genetic overlap between them. For example, a significant genetic correlation of suicide attempt with insomnia has been reported^35^, and we have detected insomnia to also correlate with broad sense self-harm. The existence of GWAS summary statistics for insomnia^65^ makes this hypothesis testable using the approach implemented herein.

Some limitations relevant to this study must be acknowledged. First, this study focused only on a sample of European Ancestry, an approach that allows to avoid biases due to population stratification. However, this hinders our ability to extrapolate to other populations. Furthermore, the samples used in this study were mostly comprised of adults in their late 50 s for the UK-B or in their 40 s for the Australian sample. This limits our ability to understand adolescent and childhood related self-harm. This is important because children and adolescents present a higher prevalence of self-harm compared to adults. Moreover, self-harm is highly complex and heterogeneous^93^, comprising a variety of acts such as physical injury and poisoning. Additionally, the self-reported nature of the phenotypes assessed in this study, could be affected by participant specific recall bias, which would bias our results towards the null. In the present study, we modelled a broad self-harm liability regardless of suicidal intent and performed analyses to unveil its underlying aetiology. Several traits, such as depression and other psychiatric disorders are associated with an increased risk for self-harm, as evidenced by the high genetic correlations identified. Our findings might be potentially identifying factors related to the genetic liability to depression and psychopathology in general. Nonetheless, the fact that we identified a positive prediction after accounting for MDD-PRS, and that previous depression PRS were unable to predict NSSH in our target sample^34^ would suggest our findings to be related to self-harm.

In summary, we performed GWAS of *self-harm ideation* and *self-harm behaviour* and identified associations with two genetic loci and eleven genes. We characterized the SNP heritability and estimated genetic correlations between the two traits of interest and with a range of other psychiatric, behavioural and physiological traits. Our results suggested an association between the genetic predisposition to *broad sense* self-harm (both ideation and behaviour) with suicide thoughts. However, no genetic overlap between PRS for *self-harm ideation* and suicide attempt was detected. The PRS for *self-harm behaviour* was associated with suicide attempt but the association with NSSH did not reach statistical significance. In addition, our results support a partially common genetic aetiology for NSSH and suicide thoughts and for suicide thoughts and attempt, but no statistically significant evidence for a shared genetic aetiology between NSSH and suicidal attempt. Future studies should leverage novel statistical genetic approaches such as genomic structural equation modelling, to aid in the deconvolution of unique and shared genetic factors between suicidal and non-suicidal self-harm.

## Supplementary information

Supplementary Information.

Supplementary Information 2.
