# Supplementary material for: Genetic aetiology of self-harm ideation and behaviour
Source: Sci Rep. 2020 Jun 16;10:9713. doi: 10.1038/s41598-020-66737-9 (PMC7297971; doi:10.1038/s41598-020-66737-9)
Supplement: Supplementary file 2 — Supplementary Information 2. [file 41598_2020_66737_MOESM2_ESM.docx]

**Supplementary Materials for**

**Genetic aetiology of self-harm ideation and behaviour**

Adrian I. Campos,^1,2*^ Karin J.H. Verweij,^3^ Dixie J. Statham,^4^ Pamela A.F. Madden^5^, Dominique F. Maciejewski*,*^6^ Katrina A.S. Davis,^7,8^ Ann John,^9^ Matthew. Hotopf,^7,8^ Andrew C. Heath,^5^ Nicholas G. Martin^1^ and Miguel E. Rentería^1,2*^

1. Department of Genetics & Computational Biology, QIMR Berghofer Medical Research Institute, Brisbane, QLD 4006, Australia

2. Faculty of Medicine, The University of Queensland, Herston, QLD, Australia

3. Department of Psychiatry, Amsterdam UMC, University of Amsterdam, Meibergdreef 9, 1105 AZ Amsterdam, the Netherlands

4. Discipline of Psychology, School of Health and Life Sciences, Federation University, Ballarat, VIC 3550, Australia

5. Department of Psychiatry, Washington University School of Medicine, St Louis, MO 63110, USA

6. Department of Developmental Psychopathology, Behavioural Science Institute, Nijmegen, the Netherlands

7. KCL Institute of Psychiatry, Psychology and Neuroscience, London, UK

8. South London and Maudsley NHS Foundation Trust, London, UK

9. HDRUK, Swansea University Medical School, Swansea, UK.

***Correspondence:**

Adrian I. Campos (adrian.campos@qimrberghofer.edu.au) & Miguel E. Rentería (miguel.renteria@qimrberghofer.edu.au)

# Supplementary Figures


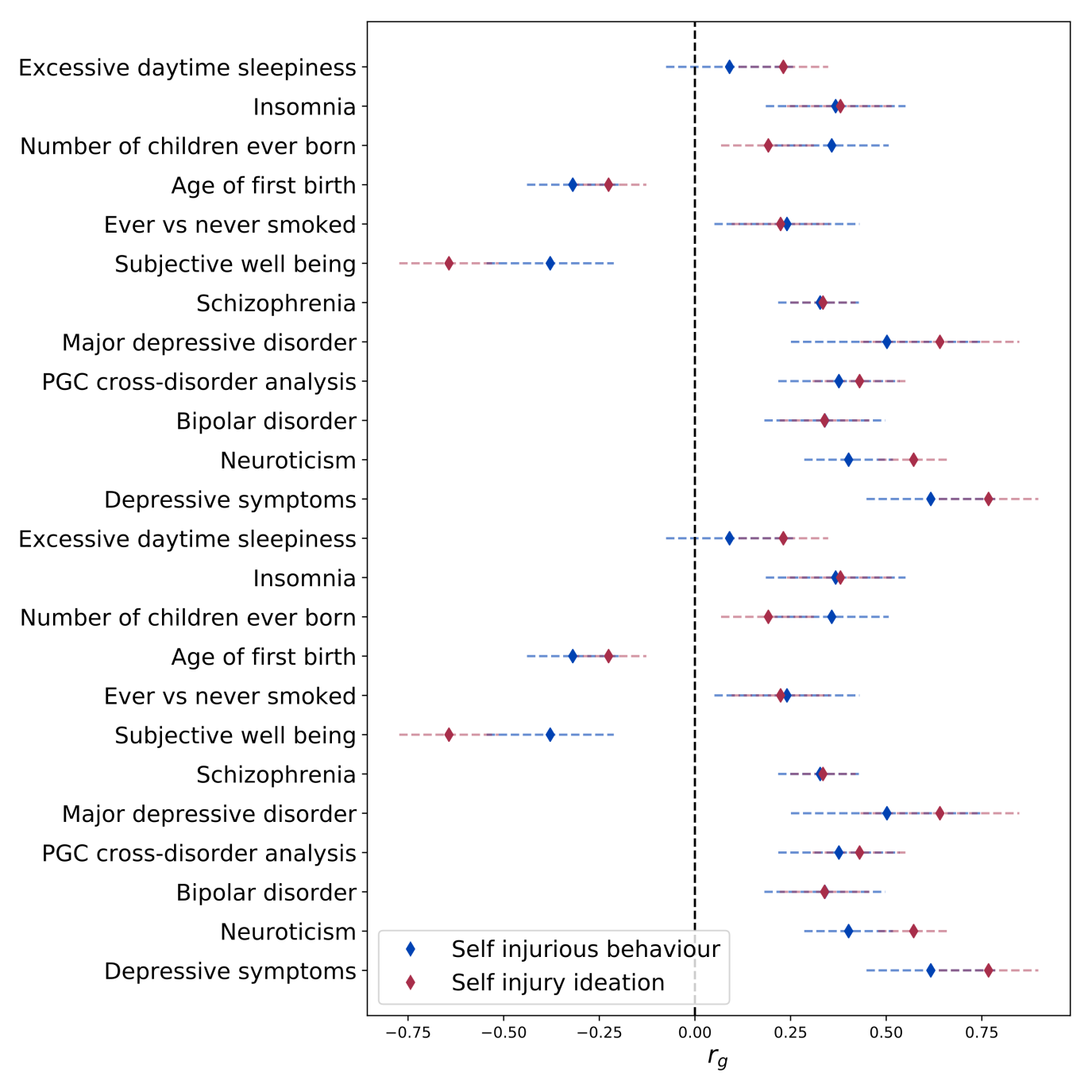


**Supplementary Figure 1.** Genetic correlations of self-injury ideation and behaviour.

Forest plot depicting the genetic correlation estimate (r_g_) and 95% confidence intervals for self-injurious behaviour (in blue) and self-injury ideation (red). Note the overall similar patterns across traits.


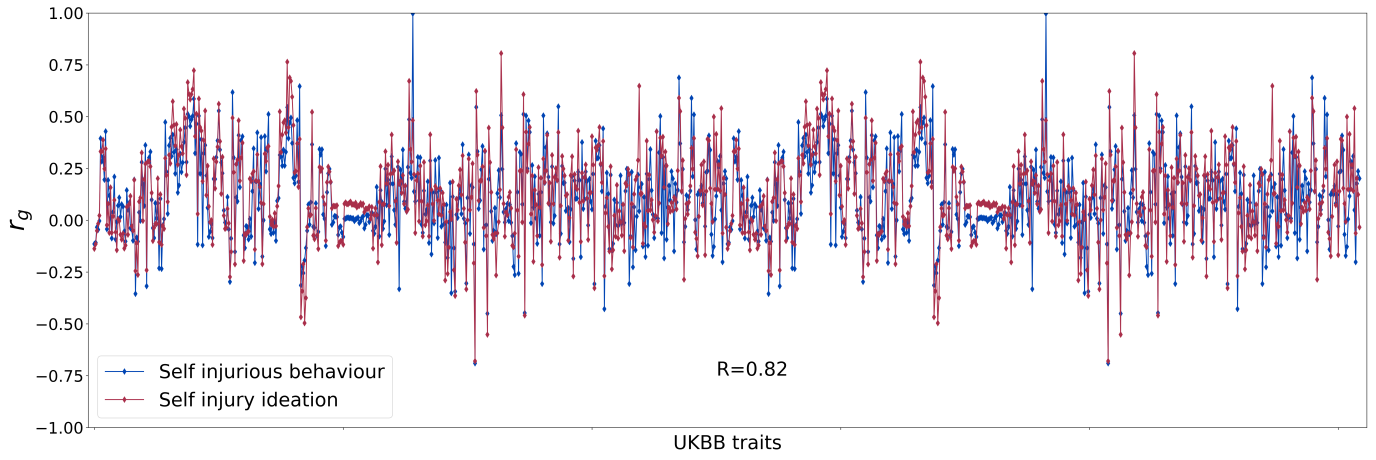


**Supplementary Figure 2.** Genetic correlations of self-injury ideation and behaviour are consistent across the UKBB traits. The x axis represents a range of traits for which summary statistics are available and have been uploaded to LD-Hub. The genetic correlations of each of these traits with our studies phenotypes are shown. Details and statistical significance are provided on the **Supplementary Table 1**.


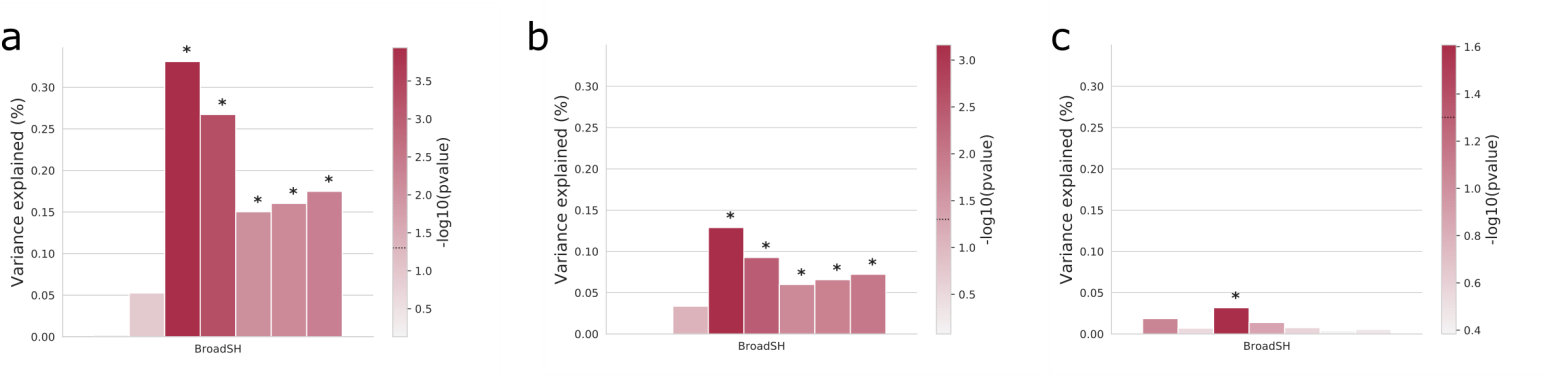


**Supplementary Figure 3. Genetic prediction of self-injurious behaviour on the target sample.**

Bar plots represent the amount of variance explained by the polygenic risk scores. a) PRS for self-injurious behaviours was significantly associated with self-injury in our target sample; PRS for self-injurious behaviours showed a diminished association after correcting for NSSI (b), and to a greater extent after correcting for suicide attempt (c).


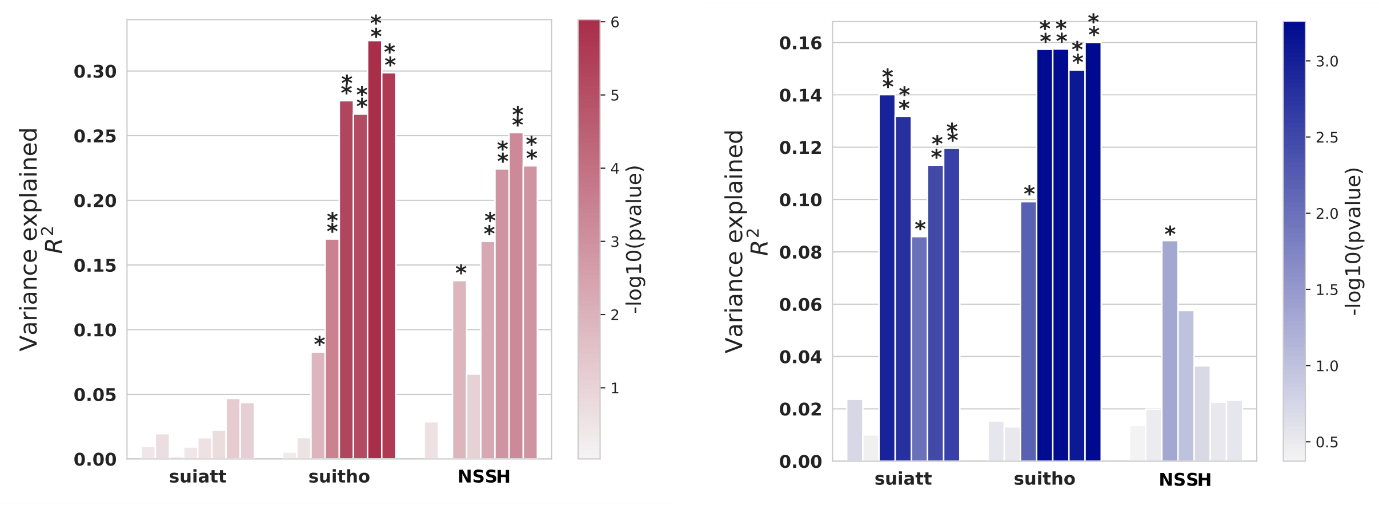


**Supplementary Figure 4. Genetic prediction of self-injurious behaviour after accounting for MDD-PRS as a covariate**

Bar plots represent the amount of variance explained by the polygenic risk scores on the self-harm phenotypes. The red colour (left side) shows the associations of the PRS for self-harm ideation whereas the blue colour (right side) depicts the associations of the PRS for self-harm behaviour. In both cases, the MDD-PRS was included as a covariate. For each phenotype studied, the amount of variance explained by a PRS including variants with an increasingly liberal p-value threshold (from left to right) is shown. The bars are ordered based on the p-value cut-off used to construct the PRS (increasingly liberal p-values). The height of each bar represents the amount of variance explained. The p-value for the association between the PRS and the phenotype is shown with a colour scale. The colour-bar legend shows a dashed black line representing the Bonferroni corrected significance threshold (α<0.05 also shown as * for simplicity).

**Supplementary Tables**

| Supplementary Table S1 | | | |
| --- | --- | --- | --- |
| p-value cutoff | suiatt % Variance explained (p) | suitho % Variance explained (p) | NSSI % Variance explained (p) |
| 0.00000005 | 0.0332 (0.207) | 0.00334 (0.692) | 0.0207 (0.323) |
| 0.00001 | 0.0188 (0.353) | 0.0372 (0.197) | 0.000179 (0.928) |
| 0.001 | 0.0161 (0.384) | 0.0699 (0.0731) | 0.143 (0.01) |
| 0.01 | 0.00946 (0.501) | 0.17 (0.00483) | 0.0785 (0.0544) |
| 0.05 | 0.0254 (0.268) | 0.353 (4.53e-05) | 0.179 (0.00356) |
| 0.1 | 0.0351 (0.197) | 0.321 (0.000109) | 0.241 (0.000779) |
| 0.5 | 0.075 (0.0585) | 0.45 (4.51e-06) | 0.271 (0.000362) |
| 1 | 0.0711 (0.0656) | 0.427 (7.96e-06) | 0.244 (0.000717) |
| Complete results of polygenic prediction using the PRS of self-harm ideation showing % of variance explained and p-values | | | |

| Supplementary Table S2 | | | |
| --- | --- | --- | --- |
| p-value cutoff | Suiatt % Variance explained (p) | Suitho % Variance explained (p) | NSSI % Variance explained (p) |
| 0.00001 | 0.0118 (0.452) | 0.0135 (0.427) | 0.017 (0.371) |
| 0.001 | 0.0499 (0.127) | 0.0218 (0.318) | 0.0174 (0.372) |
| 0.01 | 0.206 (0.0018) | 0.0785 (0.0569) | 0.0895 (0.0413) |
| 0.05 | 0.173 (0.00424) | 0.0986 (0.0327) | 0.059 (0.0974) |
| 0.1 | 0.117 (0.0183) | 0.112 (0.0224) | 0.0384 (0.18) |
| 0.5 | 0.122 (0.0155) | 0.127 (0.015) | 0.0218 (0.311) |
| 1 | 0.127 (0.0137) | 0.127 (0.0147) | 0.023 (0.298) |
| Complete results of polygenic prediction using the PRS of self-harm showing % of variance explained and p-values. No variants with p<5e-8 existed for this GWAS | | | |
